# Screening and Characterization of Multidrug-Resistant *Enterobacterales* among Hospitalized Patients in the African Archipelago of Cape Verde

**DOI:** 10.3390/microorganisms10071426

**Published:** 2022-07-14

**Authors:** Samanta Freire, Teresa Grilo, Maria Luísa Teixeira, Euclides Fernandes, Laurent Poirel, Marta Aires-de-Sousa

**Affiliations:** 1 Laboratory of Molecular Biology, Portuguese Red Cross, 1600-680 Lisboa, Portugal; samanta.c.freire@gmail.com (S.F.); teresa.grilo@emergenciacvp.pt (T.G.); 2 Hospital Universitário Agostinho Neto, Praia 112, Cape Verde; marial.teixeira@han.gov.cv (M.L.T.); euclides.fernandes@han.gov.cv (E.F.); 3 Medical and Molecular Microbiology Unit, Faculty of Science and Medicine, University of Fribourg, 1700 Fribourg, Switzerland; laurent.poirel@unifr.ch; 4 INSERM European Unit (IAME, France), University of Fribourg, 1700 Fribourg, Switzerland; 5 Swiss National Reference Center for Emerging Antibiotic Resistance (NARA), 1700 Fribourg, Switzerland; 6 Escola Superior de Saúde da Cruz Vermelha Portuguesa-Lisboa (ESSCVP-Lisboa), 1300-125 Lisboa, Portugal; 7 Laboratory of Molecular Genetics, Instituto de Tecnologia Química e Biológica António Xavier (ITQB), Universidade Nova de Lisboa (UNL), 2780-157 Oeiras, Portugal

**Keywords:** ESBL, *Enterobacterales*, *Klebsiella pneumoniae*, *Escherichia coli*, Africa, Cape Verde

## Abstract

This study aimed to investigate, for the first time, the occurrence and characteristics of extended-spectrum β-lactamase (ESBL)- and carbapenemase-producing *Enterobacterales* in Cape Verde. A total of 98 inpatients hospitalized at Hospital Universitário Agostinho Neto were screened for rectal colonization. All ESBL- and carbapenemase-producing isolates were tested for antimicrobial susceptibility and characterized by multilocus sequence typing. Mating-out assay followed by PCR-based replicon typing were performed to characterize the plasmids harboring carbapenemase encoding genes. A large proportion of patients carried ESBL- or carbapenemase-producing *Enterobacterales* (56% and 6%, respectively). Among 93 ESBL-producing isolates, there were mainly *Klebsiella pneumoniae* (58%) and *Escherichia coli* (37%). Five different ESBLs were detected, with CTX-M-15 being highly predominant (92%). Six carbapenemase-producing isolates (five *E. coli* and one *K. pneumoniae*) were recovered, and all of the OXA-48-like type (four OXA-181, one OXA-48, and one OXA-244). The *bla*_OXA-48_ gene was located on an IncFI-type plasmid, the *bla*_OXA-181_ gene on IncFI or IncX3 plasmids, and the *bla*_OXA-244_ gene was found to be chromosomally located. The five carbapenemase-producing *E. coli* isolates belonged to five distinct sequence types. This study overall showed a very high prevalence of ESBL-producing *Enterobacterales*, as well as the emergence of carbapenemase producers in this hospital.

## 1. Introduction

Carbapenem-resistant and extended-spectrum β-lactamase (ESBL)-producing *Enterobacterales* are critical antimicrobial-resistant bacteria on the World Health Organization priority list to guide the research, discovery, and development of new antimicrobials [1]. The emergence of ESBL- and carbapenemase-producing Gram-negative bacteria and their subsequent spread is considered a global threat, including resource-poor regions such as the Portuguese-speaking African countries (PALOP).

Among PALOP countries, a prospective study conducted in 2015 in a pediatric hospital in Angola showed that 36% of the screened individuals were found to be positive for carbapenemases, and 88% of those carbapenemase-producing isolates harbored the *bla*_OXA-181_ gene [2]. Two years later, the rate of colonization by carbapenemase-producing *Enterobacterales* doubled (78%) and the emergence of NDM-like carbapenemases was noticed among half of the isolates. This showed an alarming increase and evolution of carbapenemase producers in this hospital [3]. In the archipelago of São Tomé and Principe, the rate of colonization by carbapenem-resistant *Enterobacterales* among patients was 44%, and all produced the same carbapenemase, OXA-181 [4]. In Cape Verde, a single study evaluated the antimicrobial susceptibility among *Escherichia coli* isolates between 2013 and 2017, and showed increasing resistances for ceftriaxone and cefuroxime, but the resistance mechanisms were not investigated [5].

The aim of the present study was therefore to investigate the occurrence and genetic characteristics of ESBL- and carbapenemase-producing *Enterobacterales* colonizing patients in a hospital of Cape Verde.

## 2. Materials and Methods

### 2.1. Bacterial Isolates and Susceptibility Testing

A total of 98 rectal swabs were collected in October/November 2021 from 98 patients hospitalized for >48 h at Hospital Universitário Agostinho Neto (HUAN) in the island of Santiago, Cape Verde. Samples were collected from different wards: surgery (n = 30), orthopedics (n = 30), internal medicine (n = 28), and emergency room (n = 10). The samples were incubated overnight in 3 mL of Tryptic Soy Broth (TSB) (Frilabo, Maia, Portugal) for enrichment. The next day, a volume of 25 μL of each broth was inoculated onto three selective media, according to the manufacturer’s recommendations: (i) CHROMagar ESBL (ChromAgar, Paris, France) to select for ESBL producers; (ii) CHROMagar mSuperCARBA (ChromAgar) to select for carbapenem-resistant isolates; and (iii) CHROMagar COL-APSE (ChromAgar) to select isolates resistant to colistin. The isolates that were selected in the different media were identified at the species level using the API20E system (bioMérieux, La Balme-les-Grottes, France) or by MALDI-TOF in the case of doubtful results.

Antimicrobial susceptibility testing was performed using the disc diffusion method on Mueller–Hinton (MH) agar plates (Neogen, Lansing, Michigan) for ticarcillin, amoxicillin/clavulanic acid, cefotaxime, ceftazidime, temocillin, cefoxitin, ceftazidime/avibactam, ertapenem, imipenem, meropenem, aztreonam, ciprofloxacin, trimethoprim–sulfamethoxazole (SXT), tetracycline, amikacin, and gentamicin, following EUCAST recommendations.

### 2.2. Molecular Analysis

Identification of ESBL and carbapenemase genes was performed by PCR as previously reported [6,7]. Screening of the colistin resistance mcr genes (mcr-1 to mcr-9) was performed by using previously published primers [8]. All positive amplicons were sent for sequencing. Additionally, we used standard PCR conditions to amplify the β-lactamase gene bla_CMY_, encoding plasmid-mediated CMY-type cephalosporinases [9].

Clonal relationship of the carbapenemase-producing isolates was evaluated by multilocus sequence typing (MLST) [10]. Sequence types (STs) were assigned using the MLST databases for *K. pneumoniae* and *E. coli* (https://cge.cbs.dtu.dk/services/MLST-2.0/ (accessed on 12 July 2022)).

### 2.3. Conjugation Experiments and Plasmid Analysis

Mating-out assays were performed using the azide-resistant *E. coli* J53 as the recipient. *E. coli* J53 and bla_OXA-48-like_-positive donors were separately inoculated into TSB (5 mL) and incubated for 5 h. The samples were subsequently mixed at a ratio of 1:4 (200 µL donor/800 µL recipient), centrifuged at 3500 rpm for one minute, and 800 µL of the supernatant was discarded. The pellet was resuspended in the remaining 200 µL, which were deposited onto 22 µm filters and incubated for 3 h at 37 °C onto Tryptic Soy Agar (TSA) plates (Frilabo). After the incubation, filters were resuspended in 5 mL NaCl 0.9% and 100 µL of this mixture was plated onto TSA agar plates supplemented with ticarcillin (100 µg/mL) and azide (100 µg/mL). Susceptibility testing was performed for all *E. coli* transconjugants, and positivity for bla_OXA-48-like_ was assessed by PCR.

Plasmids were classified according to their incompatibility group using the PCR-based replicon typing (PBRT) method performed on DNA recovered from *E. coli* transconjugants, as described previously [11].

### 2.4. Ethical Approval

The protocol was approved by the institutional review board of the hospital. Informed consent was obtained from the patients after a verbal presentation of the purpose, method, and design of the study.

## 3. Results and Discussion

### 3.1. Population Description and Antibiotics Prescription

A total of 98 inpatients were screened. The demographic data showed that 61% of the individuals were male and 97% were adults, with a median age of 48.0 ± 17.8 years. Half of the patients (n = 49) were admitted to the hospital the week preceding sampling (between 48 h and 8 days), and the main reasons for admission were orthopedic (30%), vascular (16%), respiratory (9%), and gastrointestinal (7%). The mean number of days of hospitalization was 13.0, being the highest among the orthopedic patients (18.6 days) and the lowest in the emergency ward (9.1 days).

All but three patients received antibiotics in the seven days prior to the sampling. Cephalosporins (mainly ceftriaxone) were the antibiotics most prescribed (40 patients), alone or in combination with other antibiotics, followed by ciprofloxacin, flucloxacillin, metronidazole, and amoxicillin/clavulanic acid (Table 1). While cephalosporins were used in all wards, ciprofloxacin was mainly used in surgery and internal medicine, flucloxacillin used in orthopedics, and metronidazole and amoxicillin/clavulanic acid were almost exclusively used in internal medicine. A single patient received a carbapenem (imipenem).

### 3.2. Carriage of Multidrug-Resistant Enterobacterales

Out of the 98 patients from whom rectal swabs were taken, 97 distinct enterobacterial isolates showing a phenotypic antibiotic resistance profile, compatible with the production of ESBLs or carbapenemases, were recovered from 61 different individuals. Overall, 55 patients were colonized with an ESBL producer (56%) and six carried a carbapenemase-producing isolate (6%) (Table 2). Of note, carbapenems were not administrated to any of the latter six patients. Among the four wards included in the study, the highest rate of colonization, by both ESBL and carbapenemase producers, was observed in surgery (63% and 10%, respectively), but with no statistical significance (*p* = 0.29). No enterobacterial isolate was positive for colistin resistance *mcr* genes.

### 3.3. ESBL-Producing Enterobacterales

Among the 55 patients colonized by ESBL-producing *Enterobacterales*, 22 carried more than one type of ESBL-producing isolate, leading to a total of 93 isolates (Table 3). Three species were identified: *K. pneumoniae* (n = 54; 58%), *E. coli* (n = 34; 37%), and *Enterobacter cloacae* (n = 5; 5%).

The genotypic characterization identified five different ESBLs (Table 3), all but one being of the CTX-M-type (99%); SHV-2 was detected in a single *K. pneumoniae* isolate. The most commonly identified ESBL was CTX-M-15 (n = 86; 92%). ESBLs belonging to CTX-M group 9 were exclusively identified in *E. coli*, namely CTX-M-14 (n = 4), CTX-M-9 (n = 1), and CTX-M-129 (n = 1). Two *E. coli* isolates producing CTX-M-15 co-harbored CMY-type β-lactamase-encoding genes, and two other isolates coproduced a carbapenemase.

Notably, several ESBL-positive rectal samples carried different bacterial species or different ESBLs. Nineteen (35%) out of the 55 carriers of ESBL-producing *Enterobacterales* were colonized by two or more species (*K. pneumoniae/E. coli* (n = 15); *E. coli/E. cloacae* (n = 2); *K. pneumoniae/E. coli/E. cloacae* (n = 2)). Three rectal samples contained two different ESBLs (CTX-M-15 and CTX-M-14).

This study investigated for the first time the acquired β-lactam resistance mechanism at the molecular level, evidencing very high rates (56%) of ESBL-producing *Enterobacterales* among inpatients in this hospital. A previous work evaluated the antimicrobial drug susceptibility among *E. coli* isolates in HUAN, and showed significant increases in resistance for ceftriaxone from 2013 to 2017 (from ~15% to ~40%), this observation being compatible with a likely increase in ESBL-producing isolates, although the resistance mechanism was not investigated [5].

### 3.4. Carbapenemase-Producing Enterobacterales

The six carbapenemase-producing isolates were identified as *E. coli* (n = 5; 83%) and *K. pneumoniae* (n = 1). All carbapenemases identified were of the OXA-48-like type (Table 4). Four isolates carried the *bla*_OXA-181_ gene, a single isolate harbored the *bla*_OXA-48_ gene, and another single isolate harbored the *bla*_OXA-244_ gene. The two latter isolates additionally co-produced an ESBL (CTX-M-15). Moreover, a single *E. coli* isolate producing OXA-181 co-produced a CMY-type AmpC. None of the OXA-181-producing *E. coli* isolates co-produced an ESBL, therefore remaining susceptible to broad-spectrum cephalosporins (except the isolate producing CMY), by contrast to the OXA-244 and OXA-48 isolates. Such rare association of the *bla*_OXA-181_ gene with an ESBL-encoding gene among *E. coli* isolates, contrasting with the frequent associations observed among *bla*_OXA-48_-positive *K. pneumoniae* isolates, in particular with the *bla*_CTX-M-15_ ESBL gene, has been previously observed among isolates from another PALOP country, Angola [2].

To the best of our knowledge, when considering the whole African continent, OXA-244-producing isolates have only been detected so far in Egypt, specifically among *E. coli* [12,13]. Of note, the single *E. coli* isolate producing OXA-244 that was identified in the present study was not recovered from the carbapenem-selective medium, but from the ESBL-selective plates as a consequence of its CTX-M-15 co-production. As previously reported, OXA-244-producing *E. coli* isolates are difficult to detect using several commercial media, due to the low carbapenemase activity of that enzyme, which often leads to the low-level MICs of carbapenems. Such misrecognition might therefore explain its low prevalence and consequently favor its silent spread [14].

Mating-out assays followed by PBRT revealed that the *bla*_OXA-48_ gene was located on an IncFI-type plasmid. This was quite unexpected since the carbapenemase gene is known to be most commonly carried by an epidemic IncL plasmid worldwide. Nevertheless, the association of *bla*_OXA-48_ with an IncFI plasmid was previously reported in India [15]. The *bla*_OXA-181_ gene was either located on IncFI or IncX3 plasmids (Table 4). Conjugation assays performed with the *E. coli* isolate producing OXA-244 remained unsuccessful, suggesting that the *bla*_OXA-244_ gene was located on the chromosome, as previously reported [12,16].

MLST analysis showed that the five carbapenemase-positive *E. coli* isolates were distributed into five different clones (Table 4). However, two isolates carrying *bla*_OXA-181_, classified as ST940 and as a triple-locus variant of ST940, belonged to a clonal complex (CC448). To date, *E. coli* isolates belonging to ST940 have been sporadically reported. The first *E. coli* ST940 isolates reported harbored the *bla*_NDM-5_ gene, encoded on IncX3 and IncFII plasmids, and caused invasive infection in an immunocompromised pediatric patient hospitalized in 2014 in Lebanon and later transferred to the USA [17]. ST940 has been also found in Ghana, in *E. coli* carrying the *bla*_OXA-181_ gene localized on an IncFIC plasmid [18], and it has been recently reported as the predominant clone among uropathogenic *E. coli* isolates in India [19].

The single carbapenemase-producing *K. pneumoniae* isolate belonged to a new ST, which is a double-locus variant of the international multidrug-resistant clone ST307. The latter ST emerged during the mid-1990s in Europe, then spread to all continents and is currently endemic in Italy, Colombia, the United States (Texas), and South Africa [20]. Moreover, it has been associated with the global dispersion of OXA-48, OXA-181, OXA-232, and OXA-204 producers [21]. In the African continent, besides South Africa, ST307 was found to be predominant among ESBL-producing *K. pneumoniae* isolated between 2016 and 2018 from three sentinel surveillance hospitals in Nigeria [22]. In addition, a *K. pneumoniae* ST307 clinical isolate harboring the *bla*_OXA-48_ gene on an IncL-type plasmid was reported in Tunisia in the same period [23]. Of note, genome sequencing identified ST307 among chimpanzees and termites in protected areas in Senegal, suggesting the circulation of strains between the two species [24].

Our study reports for the first time the emergence of carbapenemase-producing *Enterobacterales* among hospitalized patients in Cape Verde (6%). Although it is a low occurrence compared to other PALOP countries, which showed much higher rates, such as Angola (78%) and São Tomé and Principe (44%) [2,3,4], it should be a matter of concern in a country where carbapenems are still scarcely used. The extensive consumption of penicillins or the amoxicillin–clavulanic acid combination (Table 1) could be the driving factors of selection of OXA-48-like carbapenemases, which confer high-level resistance to penicillins and penicillin-β-lactamase inhibitor combinations.

### 3.5. Antimicrobial Resistance

**Figure 1:**
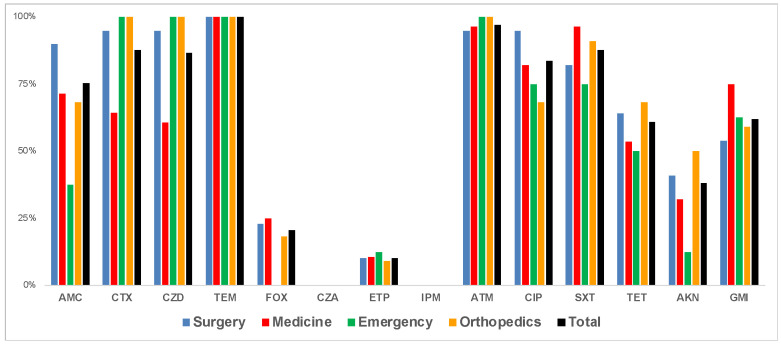
Antimicrobial resistance among the 97 ESBL- and carbapenemase-producing enterobacterial isolates, by ward. AMC—amoxicillin/clavulanic acid; CTX—cefotaxime; CZD—ceftazidime; TEM—temocillin; FOX—cefoxitin; CZA—ceftazidime/avibactam; ETP—ertapenem; IPM—imipenem; ATM—aztreonam; CIP—ciprofloxacin; SXT—trimethoprim/sulfamethoxazole; TET—tetracycline; AKN—amikacin; GMI—gentamicin.

Among the 97 enterobacterial isolates, we observed non-susceptibility against temocillin (100%), aztreonam (n = 94; 97%), cefotaxime and SXT (n = 85; 88%), ceftazidime (n = 84; 87%), ciprofloxacin (n = 81; 84%), amoxicillin/clavulanic acid (n = 73; 75%), gentamicin (n = 60; 62%), tetracycline (n = 59; 61%), amikacin (n = 37; 38%), cefoxitin (n = 20; 21%), and ertapenem (n = 11; 11%) (Figure 1). Five ESBL-producing isolates (four *K. pneumoniae* and one *E. cloacae* producing CTX-M-15) showed resistance to ertapenem, which might be due to membrane permeability defects. All ertapenem-resistant isolates remained susceptible to imipenem and meropenem. None of the isolates were resistant to the newly developed ceftazidime/avibactam combination.

Of note, although aminoglycosides and SXT were barely prescribed to the patients included in the present study (Table 1), non-susceptibility to gentamicin and SXT was found in over 60% of the isolates (Figure 1). Very high rates of SXT resistance are common in low-income countries, including African countries, where its prophylactic use is widely recommended for HIV-infected people. In addition, it is typically used in diarrhea empiric therapy [25], is inexpensive, is usually available without prescription, and therefore is commonly used.

In conclusion, this study constitutes the first report of enterobacterial ESBL and carbapenemase producers in Cape Verde, where no molecular epidemiological data were previously available, and confirms that the African continent is an important reservoir of OXA-181 producers.

## Figures and Tables

**Table 1:** Prescription of antibiotics among the 98 patients from the different wards.

Antibiotic Class	Antibiotic	Ward (Nº Patients)				
		Surgery N = 30	Orthopedics N = 30	Internal Medicine N = 28	Emergency N = 10	Total N = 98
Cephalosporins	Ceftriaxone	8	15	9	5	37
	Cefazolin	1	2			3
Fluoroquinolones	Ciprofloxacin	10	4	10		24
Penicillins	Flucloxacillin	4	17		1	22
	Ampicillin	1		1		2
	Penicillin G	2				2
Nitroimidazole	Metronidazole	4	1	10		15
Penicillin/inhibitor	Amox/CA	1		13		14
Lincosamides	Clindamycin	4				4
Chemotherapeutics	SXT			3	1	4
Macrolides	Azithromycin			2		2
Aminoglycosides	Amikacin	1		1		2
	Gentamicin		1			1
Carbapenems	Imipenem				1	1
Glycopeptides	Vancomycin		1			1

Amox/CA—amoxicillin/clavulanic acid; SXT—trimethoprim–sulfamethoxazole.

**Table 2:** Rates of colonization with ESBL or carbapenemase producers by ward.

Ward	Patients Screened	No. of Patients Colonized with ESBL Producers	No. of Patients Colonized with Carbapenemase Producers
Surgery	30	19 (63%)	3 (10%)
Orthopedics	30	16 (53%)	1 (3%)
Internal Medicine	28	14 (50%)	1 (4%)
Emergency	10	6 (60%)	1 (10%)
Totals	98	55 (56%)	6 (6%)

**Table 3:** Characteristics of the 93 ESBL-producing isolates.

Species	ESBL/AmpC	TIC	AMC	CTX	CZD	TEM	FOX	CZA	ETP	IPM	ATM	CIP	SXT	TET	AKN	GMI
*K. pneumoniae* (54)	CTX-M-15 (53) ^1^	R	R(41)	R	R	R	S(43)	S	S(48)	S	R	R(49)	R(51)	S(31)	S(35)	R(43)
	SHV-2 (1)	R	R	R	R	R	R	S	S	S	R	S	S	R	S	S
*E. coli* (34)	CTX-M-15 (26) ^1^	R	R(15)	R	R	R	S(2)	S	S	S	R	R(21)	R(19)	R(18)	S(13)	S(15)
	CTX-M-15/ CMY (2)	R	R	R	R	R	R	S	S	S	R	R	R	R	S(1)	R
	CTX-M-14 (4)	R	S(2)	R	R	R	S	S	S	S	R	S(2)	R	R	S(2)	S
	CTX-M-9 (1)	R	R	R	S	R	S	S	S	S	R	R	R	R	R	S
	CTX-M-129 (1)	R	S	R	R	R	S	S	S	S	R	R	R	R	S	S
*E. cloacae* (5)	CTX-M-15/ CMY (5)	R	R	R	R	R	R	S	S(4)	S	R	R	R(4)	R	R(4)	R

^1^ Two CTX-M-15-producing isolates (1 *E. coli* and 1 *K. pneumoniae*) coproduced a carbapenemase, and the antibiotic resistance is displayed exclusively in Table 4. Numbers within parentheses indicate the number of isolates that are resistant or susceptible to the antibiotic, if not all. Resistance is shown in grey shade for better visualization relative to susceptibility with no grey shade. TIC—ticarcillin; AMC—amoxicillin/clavulanic acid; CTX—cefotaxime; CZD—ceftazidime; TEM—temocillin; FOX—cefoxitin; CZA—ceftazidime/avibactam; ETP—ertapenem; IMP—imipenem; ATM—aztreonam; CIP—ciprofloxacin; SXT—trimethoprim–sulfamethoxazole; TET—tetracycline; AKN—amikacin; GMI—gentamicin.

**Table 4:** Characteristics of the 6 carbapenemase-producing isolates.

Species	Isolate	Patient	ST	CC	Carbapenemase	ESBL/AmpC	Plasmid Type ^1^	TIC	AMC	CTX	CZD	TEM	FOX	CZA	ETP	IPM	MEM	ATM	CIP	SXT	TET	AKN	GMI
*E. coli*	H9CARB R	9	940	448	OXA-181		IncFI	R	R	S	S	R	S	S	R	S	S	S	S	S	R	S	S
*E. coli*	H21CARB R	21	1196	ND	OXA-181		IncX3	R	R	S	S	R	S	S	R	S	S	S	R	S	R	S	S
*E. coli*	H29CARB R	29	410	23	OXA-181	CMY	IncX3	R	R	R	R	R	R	S	R	S	S	R	R	R	R	S	S
*E. coli*	H48CARB R	48	TLV 940	448	OXA-181		IncFI	R	R	S	S	R	S	S	R	S	S	S	S	R	S	S	S
*E. coli*	H61E R	61	DLV 69	69	OXA-244	CTX-M-15	-	R	R	R	I	R	S	S	R	S	S	I	S	S	S	S	S
*K. pneumoniae*	H88E A	88	DLV 307		OXA-48	CTX-M-15	IncFI	R	R	R	R	R	S	S	R	S	S	R	S	R	R	S	S

ST—sequence type determined by multilocus sequence typing. DLV—double-locus variant; TLV—triple-locus variant. CC—clonal complex. TIC—ticarcillin; AMC—amoxicillin/clavulanic acid; CTX—cefotaxime; CZD—ceftazidime; TEM—temocillin; FOX—cefoxitin; CZA—ceftazidime/avibactam; ETP—ertapenem; IMP—imipenem; MEM—meropenem; ATM—aztreonam; CIP—ciprofloxacin; SXT—trimethoprim–sulfamethoxazole; TET—tetracycline; AKN—amikacin; GMI—gentamicin. ^1^ Plasmid carrying the carbapenemase gene.

## Data Availability

The data presented in this study are all available in the main text.
